# Secretion of cochlin-tomoprotein (LCCL) in the middle ear following acute tympanic injury: implications for perilymph fistula diagnosis

**DOI:** 10.3389/fneur.2025.1527311

**Published:** 2025-04-02

**Authors:** Seong Hoon Bae, Tetsuo Ikezono, Haeng Ran Park, Hyoyeol Kim, Tomohiro Matsumura, Shiho Saito, Yukihide Maeda, Han Matsuda, Jinsei Jung

**Affiliations:** ^1^Department of Otorhinolaryngology, Gangnam Severance Hospital, Yonsei University College of Medicine, Seoul, Republic of Korea; ^2^Institute for Lee Won Sang Yonsei Ear Science, Seoul, Republic of Korea; ^3^Department of Otorhinolaryngology, Faculty of Medicine, Saitama Medical University, Saitama, Japan; ^4^Department of Otorhinolaryngology, Brain Korea 21 PLUS Project for Medical Sciences, Yonsei University College of Medicine, Seoul, Republic of Korea; ^5^Isotope Research Laboratory, Nippon Medical School, Tokyo, Japan

**Keywords:** cochlin tomoprotein, LCCL, perilymph, middle ear, tympanostomy

## Abstract

**Introduction:**

Cochlin is the most abundant protein in the inner ear. The cleaved N-terminal domain of cochlin, known as LCCL and referred to as CTP (cochlin tomoprotein) in clinical biomarker usage, is a perilymph-specific protein and is widely used as a biomarker to detect perilymph leakage. However, little is known about the secretion or presence of LCCL in the middle ear, even though it can result in false positives when using LCCL as a biomarker for perilymph leakage.

**Methods:**

We conducted translational research in humans and mice. A retrospective observational study was conducted on human patients who underwent multiple CTP tests after tympanostomy. In parallel, an experimental study on cochlin and its cleaved product, LCCL, was performed in mice.

**Results:**

We found the exceptionally elevated level of CTP within 10 days after tympanostomy in humans regardless of the presence of definite perilymph leakage. In addition, we identified LCCL in the middle ear after tympanostomy in mice. The concentration of LCCL peaked at three days post-tympanic injury. Importantly, the origin of LCCL in the middle ear lavage was not from the inner ear but is secreted from the middle ear space especially the annular ligament, suggesting it functions as an innate immune response in the middle ear.

**Conclusion:**

Tympanostomy for the CTP test results in a false positive when the sampling is delayed. While LCCL is a reliable biomarker for clinically detecting perilymph fistula, the timing of its application should be carefully considered to avoid false-positive results.

## Background

Cochlin, encoded by the *COCH* gene, is implicated in autosomal dominant nonsyndromic hearing loss, particularly in the DFNA9 subtype (1–4). The Limulus factor C, Cochlin, and Lgl1 (LCCL) domain, located at the N-terminus of cochlin, regulates innate immunity across various organ systems, including the inner ear (5–10). During inflammatory processes, aggrecanase-1–an enzyme upregulated in these conditions–cleaves the LCCL peptide from cochlin, facilitating its release from the spleen to targeted tissues (7, 9, 11). Cochlin is the predominant protein in the inner ear, where the blood-labyrinth barrier limits the infiltration of immune cells from systemic circulation (5, 6). It plays a crucial role in modulating immune responses in the inner ear by releasing the LCCL peptide into intra-cochlear compartments, such as the spiral ligament and spiral limbus (2, 3, 5). Previous studies have consistently detected LCCL in the perilymph under physiological conditions (5, 6, 12), with significant upregulation in response to inflammatory insults such as bacterial labyrinthitis and noise-induced cochlear injury in animal models (5, 6, 13).

Additionally, the LCCL peptide is an established biomarker for diagnosing perilymphatic fistula (PLF)–an aberrant communication between the inner ear’s fluid-filled compartments and air-filled structures of the middle ear, mastoid, or intracranial cavities in humans (12, 14, 15). We previously identified the cochlin-tomoprotein (CTP) isoform of LCCL as a perilymph-specific protein, with no detectable presence in blood, cerebrospinal fluid, or saliva (16). Furthermore, we characterized the diagnostic sensitivity and specificity of CTP using enzyme-linked immunosorbent assay (CTP–ELISA) of the middle ear lavage (MEL), elucidating its clinical utility and relevance in PLF (17–21). This assay received regulatory approval from the Japanese Food and Drug Administration and became eligible for national insurance coverage in Japan by 2022. The CTP diagnostic marker has been utilized, and a prospective study demonstrated that 20% of sudden deafness cases were CTP-positive, particularly among elderly patients and those with severe hearing loss. Notably, PLF was identified as one of the most significant causes of acute hearing loss (22). Furthermore, PLF repair surgery was shown to be effective in alleviating vestibular symptoms when appropriately diagnosed with CTP test (23). Thus, the perilymph leakage biomarker CTP has been demonstrated to be clinically useful, providing new insights into neurotology. However, since CTP is a newly discovered protein biomarker, we should exercise caution regarding its diagnostic accuracy. Cochlin expression is also observed in middle ear structures such as the ossicular joints and annular ligaments (24, 25). Therefore, it is essential to exclude potential LCCL secretion from the middle ear when interpreting CTP–ELISA results, in the context of PLF diagnosis. Consequently, this study aimed to investigate the presence and implications of the LCCL peptide in the middle ear following tympanostomy and evaluate its potential impact on the accuracy of CTP–ELISA as a diagnostic tool for PLF.

Our findings demonstrated a marked elevation in CTP levels within 10 days post-tympanostomy in humans. Similarly, murine models revealed LCCL secretion into the middle ear following acute tympanic membrane (TM) perforation, which may explain false-positive CTP results in clinical settings. Immunohistochemical analysis confirmed that the CTP detected in MEL likely originates from cochlin within middle ear structures, such as the annulus. To our knowledge, this is the first study to document increased LCCL levels in the middle ear following tympanostomy in both humans and murine models, underscoring the importance of cautious interpretation of CTP–ELISA results in suspected PLF cases.

## Methods

### Human subjects

This retrospective observational study included a review of the medical records of patients who visited the Department of Otorhinolaryngology, Saitama Medical University Hospital between May 2014 and June 2016. Inclusion criteria were (1) patients aged ≥13 years (2), suspected PLF cases subjected to CTP testing via MEL, and (3) patients with an initial negative CTP test who subsequently underwent two or more additional tests, regardless of the intervals between them. The study protocol was approved by the Institutional Ethical Review Board of Saitama Medical University (approval #13–086; Principal Investigator: Tetsuo Ikezono) and was conducted in accordance with the principles outlined in the Declaration of Helsinki.

### CTP detection ELISA test

The middle ear was irrigated with 0.3 mL saline using the MEL technique for CTP detection (26). Following CO_2_ laser-assisted myringotomy, lavage samples were centrifuged at 1250 *g* for 1 min, and the supernatants were stored at −80°C. Samples were subsequently sent to SRL, Inc. (Tokyo) for CTP quantification using a standardized human CTP (hCTP) ELISA protocol (17). Repeat myringotomy was performed for patients with healed TMs at the time of subsequent testing. The lower limit of detection for the ELISA was 0.2 ng/mL, with undetectable levels recorded as 0.2 ng/mL. CTP levels ≥0.8 ng/mL were considered positive, as previously described (17).

### Indication for CTP testing

We have determined the diagnostic accuracy of the test in the clinical setting using surgically created PLF, i.e., cochlea implantation surgery (17). We defined the diagnostic criteria as 0.8≦CTP (ng/ml) positive in the clinical usage of the CTP-ELISA, and sensitivity and specificity were 86.4 and 100%, respectively. We tested the expression specificity of the CTP by testing blood and CSF samples. The concentration was below the detection limit (0.2 ng/mL) in 38 of the 40 blood, and 14 of the 19 CSF samples. The CTP test may yield a negative result if the perilymph leakage is too small in volume, intermittent, or has naturally ceased.

The indication for CTP testing in PLF-suspected cases was based on the Japanese diagnostic criteria (27). In brief, perilymphatic fistula (PLF) should be suspected in patients presenting with hearing impairment, tinnitus, aural fullness, or vestibular symptoms, particularly when preceded by specific events. These include pre-existing middle or inner ear diseases, surgeries, head trauma (Category 1), or barotrauma—either external (Category 2; e.g., blast exposure, diving, flying) or internal (Category 3; e.g., nose-blowing, sneezing, straining). In idiopathic cases (Category 4), where no identifiable trauma or barotrauma can be recalled, diagnosis is particularly challenging. This category is currently under investigation by the Japanese PLF Study Group to establish clinically reasonable criteria for suspecting PLF. In this study period, PLF is suspected in cases where vertigo persists following sudden sensorineural hearing loss or when hearing loss fluctuates over time and gradually worsens. We excluded middle ear inflammatory conditions that could potentially affect CTP measurements, such as viral or bacterial infections. The CTP test was not indicated for cases with inner ear diseases of known etiology, such as genetic disorders, vestibular schwannoma, tumors, ototoxic drug exposure, or accompanying systemic autoimmune diseases.

### Sequential CTP testing

The percentage of positive CTP results from the first, second, and third MEL tests was calculated from patient records. Given that ELISA results from the external laboratory (SRL Inc.) had a turnaround time of 3–6 weeks, subsequent tests were conducted without knowledge of prior outcomes. Multiple tests were performed to capture intermittent or subclinical perilymph leakage, necessitating repeated evaluations for accurate diagnosis. The percentage of positive second CTP tests in patients with an initial negative test and the percentage of positive third CTP tests in patients with consecutive negative tests were determined. From the data obtained, 46.3% of second CTP tests yielded positive results following an initial negative test. The correlation between the CTP ratio (second to first test) and time (in days) between tests was analyzed using Spearman’s rank correlation, with statistical significance set at *p* < 0.05.

### Animal models

Cochlin knockout mice (C57B6 background) were obtained from Jackson Laboratory (strain: B6.129S1(Cg)-Cochtm1.1Stw/YuanJ), while wild-type C57B6 mice were sourced from Central Laboratory Animals Inc. (Seoul, Korea). Mice were housed under specific pathogen-free conditions at the animal facility in Yonsei University. All experimental protocols were approved by the Animal Ethics Committee of Yonsei University College of Medicine (approval #2023–0027). Mice aged 5–12 weeks were used in this study, with all experiments conducted under general anesthesia using intraperitoneal zolazepam (100 mg/kg) and xylazine (50 mg/kg).

### Tympanic membrane perforation and MEL in mice

A TM perforation was created under general anesthesia at the anterior malleus umbo of the pars tensa using a 26-gauge needle. MEL was performed at 0, 1, 3, and 7 days post-injury. If no perforation was present at day 0, one was induced prior to MEL. MEL was performed on the perforated side in cases of pre-existing perforation. A 30-gauge Hamilton syringe (Hamilton, Nevada, USA) was used to instill 5 μL phosphate-buffered saline (PBS) into the middle ear, followed by immediate aspiration, repeated three times to collect 15 μL of lavage fluid. The mice were euthanized after MEL. A total of 16 mice (seven ears) were used per time point.

### Western blot analysis

Western blot analyses were performed using MEL, perilymph, and annular tissue lysates, as previously described (28). Normalization with housekeeping proteins was not feasible due to the absence of cellular components in MEL and perilymph. Each MEL sample (10 μL) was mixed with the sample buffer, while 0.2 μL of perilymph was aspirated via round window puncture using a glass pipette and diluted in 10 μL of PBS before adding sample buffer. Annular lysates were prepared by carefully isolating the bulla, preserving the TM, and removing the surrounding tissues, including the auditory canal skin, ossicles, and bone components of the bulla. Lysates were processed in sodium dodecyl sulfate (SDS) lysis buffer, separated via polyacrylamide gel electrophoresis (PAGE), and transferred to a nitrocellulose membrane. The membrane was incubated with primary antibodies against the N-terminal cochlin (Millipore, #9A10D2) and C-terminal cochlin (custom-designed by Dr. Matsumura, Nippon Medical School, Japan), using antigen peptide sequences from human cochlin residues 527–544 (TGLEPIVSDVIDVIRGICRDF), which are 100% identical to the mouse and rat cochlin sequence. The C-terminal was produced as a rabbit polyclonal antibody by Sigma-Aldrich Japan G.K. (Tokyo, Japan). Protein bands were detected with enhanced chemiluminescence (Amersham Bioscience, Little Chalfont, UK) and band intensities from three independent blots (nine biological replicates) were quantified using ImageJ software (NIH). Beta-actin served as a loading control for tissue lysates but not for MEL and perilymph samples due to the absence of cellular content. The β-actin antibody was obtained from Santa Cruz Biotechnology (Santa Cruz, #sc-47778).

### Immunofluorescence

The presence of cochlin was detected in cryosections, whereas macrophages were visualized in whole-mounted tissues. The bulla and attached labyrinth were isolated, fixed in 4% formaldehyde at 4°C overnight, and decalcified in 10% ethylenediaminetetraacetic acid (EDTA) for 24 h. Cryosections (5 μm thick) and whole-mount samples were prepared, blocked with 10% donkey serum, and incubated with anti-cochlin (Millipore, #9A10D2) or PE-conjugated F4/80 (BD Biosciences, #565410) antibodies overnight at 4°C. Slides were mounted using a fluorescent mounting medium (Sigma-Aldrich, St. Louis, MO, USA) and analyzed via confocal microscopy. Quantification of immunofluorescence was performed using ImageJ software (NIH) with three biological replicate experiments.

## Results

### Sequential CTP testing in patients with suspected PLF

A total of 41 ears from 35 patients (13 females, aged 13–80 years, mean ± SD: 48.1 ± 18.6) suspected of having PLF who underwent at least two CTP tests were analyzed. The cases included in this analysis were 11 cases belonging to Category 1, 8 cases belonging to Category 2, 5 cases belonging to Category 3, and 11 cases belonging to Category 4. No positive CTP results were observed in the first test (0%), while 19 ears (46.3%) demonstrated positive results in the second test. The interval between the first and second tests ranged from 1 to 32 days (mean ± SD: 4.9 ± 7.6 days). Additionally, 20 ears from 16 patients (seven females, aged 23–80 years, mean ± SD: 53.0 ± 17.0 years) underwent a third CTP test, with seven ears (35.0%) testing positive. The interval between the first and third tests ranged from 2 to 106 days (mean ± SD: 13.6 ± 26.3 days). The mean CTP concentrations (ng/mL) were 0.26 ± 0.13 for the first test, 1.9 ± 3.6 for the second, and 1.5 ± 3.4 for the third.

**Figure 1:**
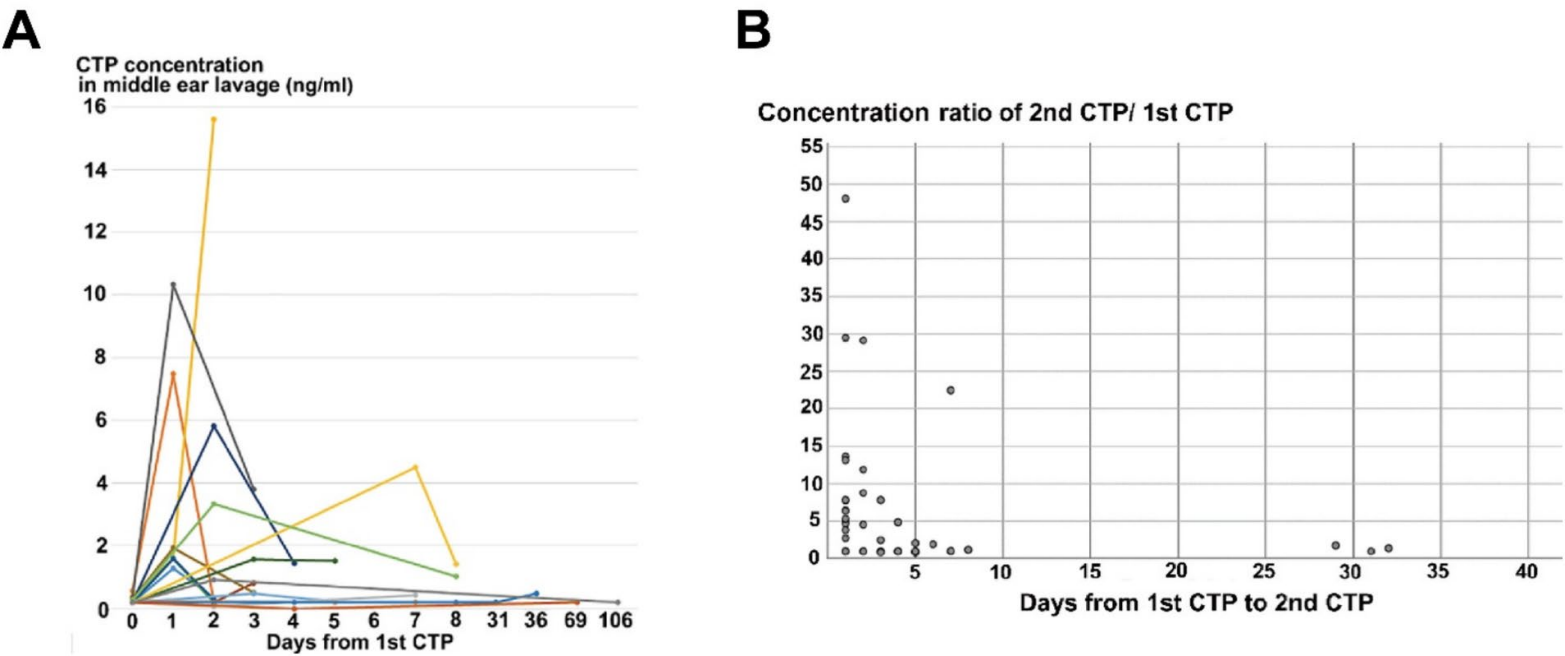
Cochlin-tomoprotein (CTP) concentration in middle ear lavage fluid from patients with suspected perilymphatic fistula (PLF). **(A)** CTP concentrations from middle ear lavage fluid in 20 ears undergoing three sequential CTP tests. The *x*-axis represents the time interval (days) between the first and either the second or third CTP test. The *y*-axis shows CTP concentrations (ng/mL). Dots connected by lines indicate sequential CTP measurements for each ear. CTP concentrations in the second test were higher than those in the first test in 14 of 20 ears, with levels returning to lower values in the third test. **(B)** Inverse correlation between the CTP concentration ratios (second/first test) and the time interval (days) between the first and second CTP tests in 41 ears. The *x*-axis shows the duration between the first and second CTP tests (days), and the y-axis shows the ratio of CTP concentrations. The correlation coefficient (*r*_s_ = −0.44, Spearman’s rank) indicates a significant inverse relationship (*p* < 0.01).

Figure 1A shows individual CTP concentrations for ears undergoing all three sequential tests, with elevated CTP levels observed in 14 of the 20 ears during the second test, followed by a reduction in the third test. Figure 1B illustrates the inverse correlation between the ratio of CTP concentrations (second to first test) and the time interval between tests (Spearman’s rank correlation coefficient, *r*_s_ = −0.44; *p* < 0.01). These findings suggest that the duration post-tympanostomy significantly influences CTP elevation after the procedure.

### LCCL presence in the middle ear following TM perforation in the mouse model

**Figure 2:**
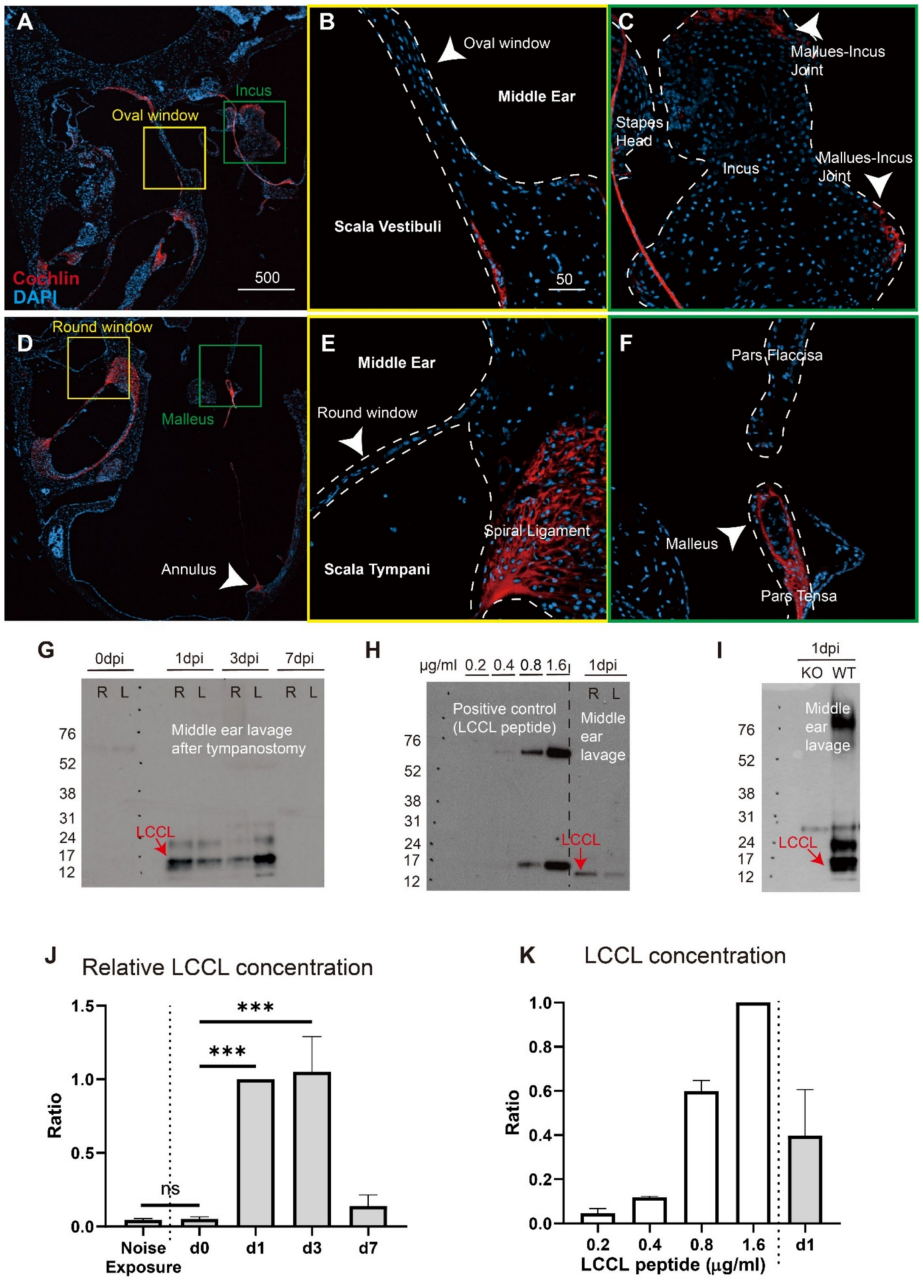
Detection of LCCL peptide in the middle ear post-tympanic membrane perforation. **(A)** Immunohistochemical localization of cochlin in the middle and inner ear structures. Cochlin is absent in the oval window (yellow box) but present in the pars tensa and incudomalleolar joint (green box). Scale bar: 500 μm. **(B)** Cochlin is absent in the magnified view of the oval window (arrowhead) in the yellow box from panel **(A)**. Scale bar: 50 μm. **(C)** Cochlin is detected in the magnified view of the incudomalleolar joint (arrowhead) in the green box from panel **(A)**. **(D)** Cochlin is absent in the round window (yellow box), but is present in the pars tensa, annulus, and tympanic membrane surrounding the malleus (green box). **(E)** Cochlin is not detected in the magnified view of the round window (arrowhead) from the yellow box in panel **(D)**. **(F)** Cochlin is present in the magnified view of the tympanic membrane and malleus (arrowhead) from the green box in panel **(D)**. Red: Cochlin; Blue: DAPI nuclear stain. **(G)** Western blot analysis of middle ear lavage fluid showing LCCL detected at 1 and 3 days post-perforation. Two samples (left and right ears) were analyzed for each time point. The vertical axis indicates molecular weight in kilodaltons (kDa). Dpi = days post-injury. **(H)** Quantification of LCCL concentration in middle ear lavage at 1 dpi (four lanes) using serial dilutions of commercial LCCL peptide as a standard. The numbers above the lanes indicate LCCL concentrations (μg/mL). The slight discrepancy in band position is due to species variation (human LCCL peptide was used as the standard). **(I)** LCCL is absent in *Coch* knockout mouse samples at 1 dpi. KO = *Coch* knockout, WT: wild-type. **(J)** Densitometric quantification of panel **(G)** shows a significant increase in LCCL concentration at 1 and 3 dpi compared to 0 dpi (seven replicates in each time point). Noise exposure data were obtained from MEL of three mice, one day after a two-hour exposure to 120 dB white-band noise. The band intensity was normalized to the 1 dpi sample. ***p < 0.001, ns: not significant. **(K)** Densitometric quantification of panel **(H)** indicates LCCL concentrations at 1 dpi ranging from 0.4 to 0.8 μg/mL. The band intensity was normalized to the 1.6 μg/mL control.

Cochlin is predominantly expressed in the inner ear, with limited documentation of its presence in the middle ear. Therefore, we performed immunofluorescence staining of middle ear sections (Figures 2A–F). Cochlin was localized in the pars tensa of the TM, the ossicular joints, and the annular ligament, consistent with previous findings by Robertson et al. (24). However, cochlin was absent from the round and oval windows (Figures 2B,E).

Subsequently, serial western blot analyses were performed on samples collected at 0, 1, 3, and 7 days post-injury (dpi) to examine the secretion of the LCCL domain into the middle ear post-TM perforation. Significantly increased concentrations of LCCL were detected at 1 and 3 dpi compared to 0 dpi (Figures 2G,J). In addition, the noise-exposed ear (120 dB white noise, 1 h) did not show LCCL in MEL. LCCL concentrations at 1 dpi ranged between 0.4 and 0.8 μg/mL, as quantified by western blot using serially diluted LCCL peptides as a reference standard (Figures 2H,K). No LCCL band was observed in the negative control at 1 dpi, although it was present in cochlin knockout mice (Figure 2I).

### LCCL secretion from the TM annulus in the mouse model

**Figure 3:**
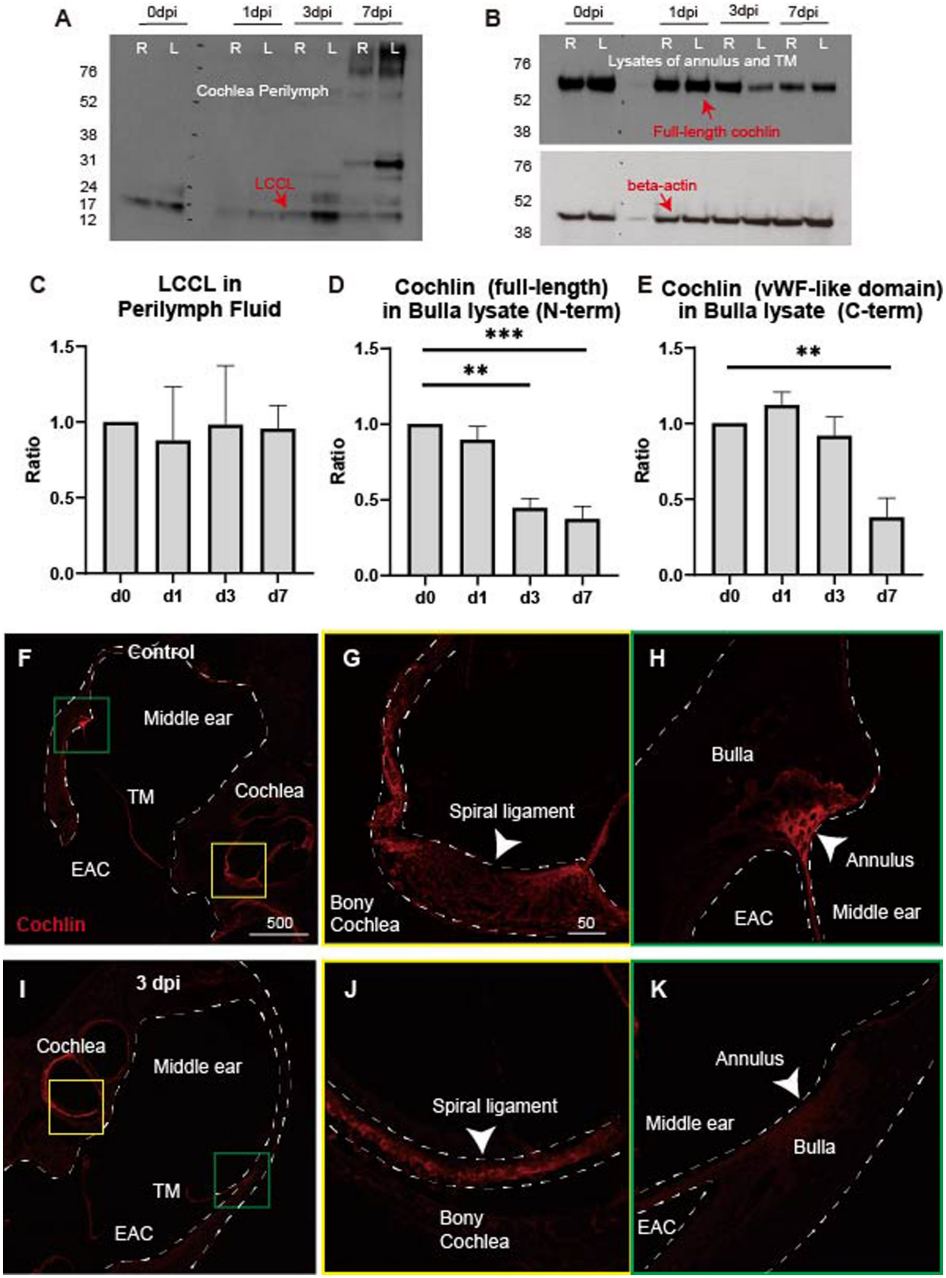
LCCL cleavage from cochlin in the annulus following tympanic membrane perforation. **(A)** LCCL is present in all perilymph samples, regardless of tympanic membrane injury, as detected by the N-terminal cochlin antibody. **(B)** Full-length cochlin in annular ligament and tympanic membrane lysates progressively decreases following tympanic membrane perforation, as detected by N-terminal cochlin antibody. Two samples (right and left) are in each time point. **(C)** Densitometric quantification of panel **(A)**, with no significant differences between groups (*n* = 4 for each time point). **(D)** Densitometric quantification of panel **(B)**, normalized to beta-actin. Full-length cochlin is significantly reduced at 3 and 7 dpi compared to 0 dpi. **(E)** Densitometric analysis of the vWFA-like domain (~40 kDa) from western analysis of bulla lysates, detected by C-terminal cochlin antibody. A significant reduction in the vWFA-like domain is observed at 7 dpi compared to 0 dpi. **p < 0.01, ***p < 0.001. **(F)** Immunofluorescence staining of cochlin using the N-terminal antibody in control inner and middle ear tissues. Strong cochlin signals are observed in the spiral ligament (yellow box) and annulus (green box). TM, tympanic membrane; EAC, external auditory canal. Scale bar: 500 μm. **(G)** A high cochlin signal is detected in the spiral ligament (arrowhead) in a magnified view of the yellow box from panel **(F)**. Scale bar: 50 μm. **(H)** Cochlin signal is similarly high in the annulus (arrowhead) in a magnified view of the green box from panel **(F)**. **(I)** Cochlin N-terminal signal decreases in the annulus (green box) but remains similar in the spiral ligament (yellow box) at 3 dpi compared to the control. **(J)** Cochlin signal remains high in the spiral ligament (arrowhead) at 3 dpi, as seen in panel **(G)**. **(K)** Cochlin signal is reduced in the annulus (arrowhead) at 3 dpi compared to panel **(H)**.

We hypothesized that the cochlin in the middle ear undergoes cleavage, leading to LCCL secretion into the middle ear space following TM injury. Therefore, we collected perilymph samples post-TM perforation and performed immunoblotting for LCCL to test whether the increased LCCL originated from the inner ear. No significant changes were observed in LCCL concentrations in perilymph samples (Figures 3A,C). Subsequently, we quantified full-length cochlin concentrations in the TM annulus before and after perforation, as this structure is known to express cochlin endogenously (24). Full-length cochlin levels were significantly decreased at 3 and 7 dpi, with both full-length and cleaved LCCL domains detected via N-terminal cochlin antibodies (Figures 3B,D). Furthermore, the von Willebrand factor A (vWFA)-like domain (~40 Da) was measured using a C-terminal cochlin antibody, revealing a significant reduction at 7 dpi compared to 0 dpi (Figure 3E).

Immunofluorescence further confirmed the reduction in full-length cochlin levels in the annulus at 3 dpi compared to controls (Figures 3F–K), without significant changes in the lateral wall of the inner ear. These data suggest that the cochlin in the TM annulus is cleaved into LCCL, which is subsequently secreted into the middle ear space. The residual cochlin, represented by the vWFA-like domain, appears to be degraded until 7 dpi.

### Inflammation of middle ear mucosa induced by tympanic membrane injury

**Figure 4:**
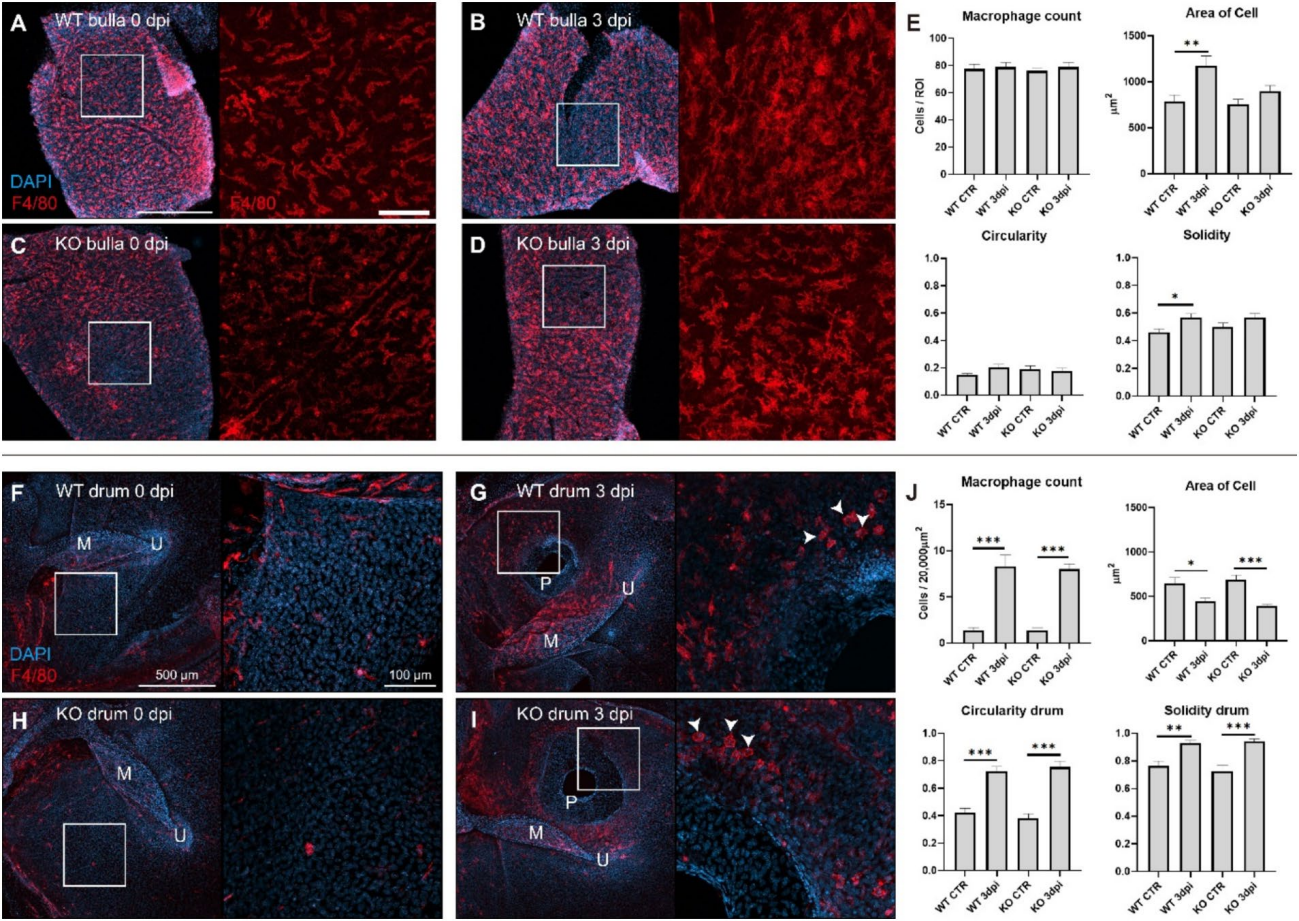
Inflammation response in the bulla mucosa and tympanic membrane following tympanic injury. **(A)** Macrophages (F4/80-positive cells, red) in the bulla mucosa of a wild-type control mouse. Scale bar: 500 μm. The right panel is a magnified view of the white box. Scale bar: 100 μm. **(B)** Macrophages in the bulla mucosa of a wild-type mouse at 3 dpi. The right panel is a magnified view of the white box, showing morphologic change of macrophage. **(C)** Macrophages in the bulla mucosa of a *Coch* knockout control mouse and a magnified view of the white box in the right panel. **(D)** Macrophages in the bulla mucosa of a *Coch* knockout mouse at 3 dpi and a magnified view of the white box in the right panel. **(E)** Quantitative analysis of panels **(A–D)** showed no change in macrophage count but an increase in cell size and solidity (higher solidity indicating a more compact cell shape). **(F)** Macrophages surrounding the tympanic membrane in a wild-type control mouse, and a magnified view of the white box in the right panel. M: malleus, U: umbo. **(G)** A tympanic membrane of a wild-type mouse at 3 dpi, showing a perforation (P) and round-shaped macrophages (white arrow head) clustering around the perforation site. A magnified view of the white box in the right panel. **(H)** A tympanic membrane of a *Coch* knockout control mouse, and a magnified view of the white box in the right panel. **(I)** A tympanic membrane of a *Coch* knockout mouse at 3 dpi, and a magnified view of the white box in the right panel, showing round-shaped macrophages around the perforation site. **(J)** Quantitative analysis of panels **(F–I)** showed significant changes in macrophage count and properties but no significant differences between wild-type and knockout mice. *P < 0.05, **P < 0.01, ***P < 0.001.

Cochlin cleavage into LCCL requires inflammation to activate the aggrecanase (5, 7); however, the inflammatory response following TM injury has not been extensively studied. Therefore, we investigated the presence of resident macrophages in the middle ear mucosa by immunostaining for F4/80-positive, a macrophage marker. Activated macrophages were observed in the mucosal layers of the middle ear bulla and TM (Figure 4). In wild-type mice, macrophages exhibited significantly increased cell size (solidity) at 3 dpi, indicative of activation in response to inflammation. Knockout mice showed a tendency but not statistically significant. In addition, the macrophage count was not increased. In contrast, the TM showed an increased number of macrophages near the perforation site after perforation, whereas fewer macrophages were present in the control samples (Figures 4F–J). In addition, these macrophages exhibited the morphology of immature macrophages transitioning from monocytes, characterized by small, circular cells. No significant differences were observed in macrophage responses between wild-type and cochlin knockout mice. These findings suggest that even minor TM injury triggers a robust inflammatory response in the middle ear mucosa, promoting cochlin cleavage and LCCL secretion into the middle ear space, paralleling observations in human tympanostomy cases.

## Discussion

Our prior identification of CTP via proteomic profiling of bovine inner ear proteins as a diagnostic marker for PLF highlights its translational clinical significance (12, 16, 17, 19–21, 26). Building on this finding, in this study, we validated the finding that LCCL–the human homolog of CTP present in perilymph–plays a pivotal role in innate immune responses within the cochlea (5, 6). Previous investigations have demonstrated that LCCL, generated through proteolytic cleavage of full-length cochlin during inflammatory cascades, augments immune activation (3, 7–9). Specifically, our data show that TM perforation induces middle ear inflammation, leading to the cochlin cleavage within the annular ligament and subsequent LCCL accumulation in MEL fluid within 1–3 dpi, followed by a gradual decline.

Clinically, perilymphatic leakage may transiently resolve following initial extravasation into the middle ear (14). However, some patients experience episodic leaks, manifesting as fluctuating auditory deficits and vertigo. These clinical observations prompted us to conduct sequential CTP assays, wherein human MEL samples demonstrated that 46% of initially CTP-negative ears in the first laser tympanostomy tested positive in the second assay. Additionally, several cases that tested positive in the second assay reverted to negative in the third assay conducted after a prolonged interval. These findings suggest that shorter inter-assay intervals, particularly those under 10 days, were associated with elevated CTP levels in the second assay.

Notably, previous studies have not detected CTP in the middle ear of patients with chronic otitis media (22, 26), indicating that CTP may be a biomarker relevant only to the early inflammatory phases of TM trauma. Thus, the role of CTP in the progression from acute to chronic middle ear inflammation warrants further investigation. In the present study, cochlin was localized to the pars tensa of the TM, annular ligament, and ossicular chain, with LCCL levels in MEL peaking 1–3 days post-TM perforation. Notably, LCCL concentrations in the perilymph remained unchanged, implying that the observed increase in MEL LCCL originated from middle ear structures. Additionally, western blot and immunohistochemical analyses revealed a reduction in full-length cochlin isoforms in the annular ligament post-perforation, suggesting that LCCL detected in MEL results from proteolytic cleavage of these isoforms. These data further indicate that positive CTP results in second MEL samples collected within 10 days of the first lavage likely reflect the TM perforation induced by the initial procedure, consistent with findings from the murine model. This phenomenon, first documented in our study, represents a biologically intriguing response, highlighting the multilayered defense and immune mechanisms mammals possess to protect auditory function. From the perspective of CTP as a diagnostic marker for perilymphatic leakage, this reaction constitutes a potential source of false-positive results. Therefore, careful interpretation of CTP–ELISA results for MEL samples collected within 10 days of tympanostomy is warranted. False-negative results in CTP testing are theoretically plausible. Even if a case is attributed to PLF, the CTP test may yield a false-negative result if the perilymph leakage is too small in volume, intermittent, or has spontaneously ceased. Such research on diagnostic biomarkers enhances scientific rigor by continuously identifying potential factors contributing to false-positive and false-negative results and refining their limitations accordingly.

Cochlin plays an integral role in cochlear innate immunity. Inflammatory insults, such as acoustic trauma or pathogen invasion, promote the release of pro-inflammatory cytokines (e.g., tumor necrosis factor-*α*, interleukin-1β, and interleukin-6), which subsequently drive the synthesis of aggrecanase-1 (29–31). Aggrecanase-1 cleaves cochlin, a key extracellular matrix component of the inner ear, releasing the LCCL domain (9, 32–34). Acute tympanic injury triggers the secretion of LCCL into the middle ear from localized sites. TM perforation also potentiates macrophage activation, as evidenced by morphological transformation of macrophages in the vicinity of the TM and bulla mucosa. However, no significant difference in macrophage activation was observed between wild-type and cochlin knockout mice. This suggests that although inflammation following TM injury elevates LCCL levels in the middle ear, LCCL does not directly modulate macrophage activation. Thus, the exact role of LCCL in middle ear pathology remains unclear, although we hypothesized that it may facilitate TM repair. Therefore, additional studies are required to elucidate this potential function.

In conclusion, cochlin is critically involved in the innate immune response of both the inner and middle ear. Following TM injury, the cleavage and subsequent release of LCCL from full-length cochlin may confound PLF diagnosis due to false-positive CTP ELISA results. Thus, caution should be exercised when interpreting CTP levels in the context of acute middle ear inflammation. Special attention should also be given to cases where traumatic PLF coincides with the middle ear or mechanical injury, such as TM perforation. Sampling for LCCL is recommended immediately after TM perforation or after at least 10 days.

## Data Availability

The original contributions presented in the study are included in the article/supplementary material, further inquiries can be directed to the corresponding authors.
